# Impaired Baroreflex Function in Mice Overexpressing Alpha-Synuclein

**DOI:** 10.3389/fneur.2013.00103

**Published:** 2013-07-23

**Authors:** Sheila M. Fleming, Maria C. Jordan, Caitlin K. Mulligan, Eliezer Masliah, John G. Holden, Ronald W. Millard, Marie-Françoise Chesselet, Kenneth P. Roos

**Affiliations:** ^1^Department of Psychology, University of Cincinnati, Cincinnati, OH, USA; ^2^Department of Neurology, University of Cincinnati, Cincinnati, OH, USA; ^3^Department of Physiology, David Geffen School of Medicine, University of California Los Angeles, Los Angeles, CA, USA; ^4^Department of Neurology, David Geffen School of Medicine, University of California Los Angeles, Los Angeles, CA, USA; ^5^Department of Neuroscience, University of California San Diego, San Diego, CA, USA; ^6^Departments of Pharmacology and Cell Biophysics, University of Cincinnati, Cincinnati, OH, USA; ^7^Department of Neurobiology, David Geffen School of Medicine, University of California Los Angeles, Los Angeles, CA, USA

**Keywords:** Parkinson’s disease, alpha-synuclein, orthostatic hypotension, baroreflex, mouse model

## Abstract

Cardiovascular autonomic dysfunction, such as orthostatic hypotension consequent to baroreflex failure and cardiac sympathetic denervation, is frequently observed in the synucleinopathy Parkinson’s disease (PD). In the present study, the baroreceptor reflex was assessed in mice overexpressing human wildtype alpha-synuclein (Thy1-aSyn), a genetic mouse model of synucleinopathy. The beat-to-beat change in heart rate (HR), computed from R–R interval, in relation to blood pressure was measured in anesthetized and conscious mice equipped with arterial blood pressure telemetry transducers during transient bouts of hypertension and hypotension. Compared to wildtype, tachycardia following nitroprusside-induced hypotension was significantly reduced in Thy1-aSyn mice. Thy1-aSyn mice also showed an abnormal cardiovascular response (i.e., diminished tachycardia) to muscarinic blockade with atropine. We conclude that Thy1-aSyn mice have impaired basal and dynamic range of sympathetic and parasympathetic-mediated changes in HR and will be a useful model for long-term study of cardiovascular autonomic dysfunction associated with PD.

## Introduction

Baroreflex failure is documented in patients with Parkinson’s disease (PD) and is often considered as the root cause for observed orthostatic hypotension (1, 2). Recently, orthostatic hypotension, baroreflex failure, and sympathetic denervation have been reported to occur early in PD, before the onset of overt motor symptoms, making cardiovascular autonomic dysfunction a potential PD biomarker for the development of disease-modifying treatments (3–7). To date few studies have reported cardiovascular autonomic dysfunction in PD mouse models and the pathologic mechanisms underlying PD-related cardiovascular autonomic dysfunction remain to be elucidated (8–11).

In PD, Lewy bodies containing alpha-synuclein have been observed in peripheral sympathetic areas including stellate ganglia and the cardiac plexus, as well as in central brainstem areas important for regulation of blood pressure and peripheral vascular resistance and compliance (12–14). Patients with a form of familial PD associated with alpha-synuclein locus triplication show impaired baroreflex-cardiovagal gain and reduced cardiac sympathetic innervation suggesting a potentially important role for alpha-synuclein in the pathogenesis of autonomic dysfunction in PD (15). Therefore, in this study we sought to characterize baroreceptor function in transgenic mice overexpressing alpha-synuclein under the Thy1 promoter [Thy1-aSyn; (16)]. These mice have increased alpha-synuclein levels in central and peripheral neurons and develop proteinase K-resistant alpha-synuclein aggregates in multiple brain regions, including the substantia nigra, locus coeruleus, and olfactory bulb (16–18). Thy1-aSyn mice display both motor and non-motor impairments including gastrointestinal and olfactory dysfunction reminiscent to that observed in PD (18–22). In addition, by 14 months of age, these mice have a significant decrease in striatal dopamine and l-DOPA responsive behavioral deficits (23). In the present study, we assessed morphometry parameters, baroreceptor reflex, and left ventricular contractility in Thy1-aSyn and wildtype (WT) mice.

## Materials and Methods

### Animals

Animal care was conducted in accordance with the United States Public Health Service Guide for the Care and Use of Laboratory Animals, and procedures were pre-approved by the Institutional Animal Care and Use Committee at the University of California Los Angeles. Transgenic mice overexpressing human alpha-synuclein under the Thy1 promoter (Thy1-aSyn) were crossed into a hybrid C57BL/6-DBA/2 background (16). Animals were maintained on the hybrid C57BL/6-DBA/2 background by breeding mutant females with male mice on the hybrid background (18, 24, 25). Littermates were never inbred. The genotype of all Thy1-aSyn and WT mice was confirmed with polymerase chain reaction (PCR) amplification analysis of DNA from tail tips at birth and confirmed at the end of the experiment. All mice were individually housed with free access to water and standard rodent chow.

Male mice from a total of 21 litters were used in this study. Litter sizes ranged from 2 to 11 mice. Separate cohorts of mice were used in the hemodynamic-baroreflex protocol (Thy1-aSyn = 9, WT = 9) and for the heart rate (HR) and contractility dobutamine challenge protocol (Thy1-aSyn = 7; WT = 7) at 9–12 months of age. An additional separate cohort of mice (Thy1-aSyn = 3–4, WT = 3–5) was implanted with arterial transducers and tested at 3–5 months of age. A younger cohort in the arterial transducer experiment was included because the survival rate in older animals was too low.

### Morphometry

Following hemodynamic assessment surgeries Thy1-aSyn and WT mice were euthanized and heart, lung, and liver weights were measured and tibia length determined.

### Hemodynamic assessment

Hemodynamic assessment was performed as previously described (26–29). Briefly, in Thy1-aSyn and WT mice anesthetized with ketamine, xylazine, and buprenorphine, both femoral arteries were catheterized with flame-stretched PE-50 tubing (30). One catheter was interfaced to a pressure transducer connected to the PC computer data acquisition system. The other catheter was used for drug injections. The functionality of the sympathetic and parasympathetic branches of the autonomic nervous system were independently evaluated by intravascular injections of the vasoconstricting α-1 adrenergic receptor agonist phenylephrine (5 and 25 μg) and the vasodilating nitric oxide donor sodium nitroprusside (5 and 15 μg). This was followed by autonomic nervous system blockers glycopyrrolate (a peripheral acting quaternary amine muscarinic receptor antagonist, 20 μg) and propranolol (β-adrenergic receptor antagonist, 50 μg) and reinfusions of phenylephrine and sodium nitroprusside to confirm abolition of the baroreceptor reflex. All drug injections were administered intrarterially in a volume of 100 μl of 0.9% aqueous saline heparinized at 7 U/ml and injected over 7 s. HR was determined from the beat-to-beat systolic pressure peak intervals.

### Dobutamine challenge

We used dobutamine to probe the integrity of the cardiac β-1 adrenergic receptor as reflected in HR and rate of left ventricular pressure change during systole (inotropic response) and diastole (lusitropic response). Thy1-aSyn and WT mice were anesthetized with ketamine, xylazine, and buprenorphine and placed on a warming pad. HR and temperature were continuously monitored during surgery. Once anesthetized, the right carotid artery was catheterized and a 1.4 Fr catheter (Millar Instruments, Houston, TX, USA) was advanced into the left ventricle to obtain a dynamic pressure signal that was mathematically differentiated into *in vivo* indices of cardiac muscle inotropy (+*dP*/*dT*) and lusitropy (−*dP*/*dT*) (26). A femoral artery was also catheterized as above for injection of dobutamine, a β-1 adrenergic receptor agonist. Dobutamine was injected in serial doses of 3, 6, 15, and 30 ng/g with at least a 5-min recovery period between injections as an adrenergic post-synaptic receptor challenge test. Measurements were acquired with Hem Software (Notocord Systems V4.2, Croissy sur Seine, France). HR and left ventricular pressure were recorded 150 s after injections and averaged over a 10-s period. Maximum and minimum *dP*/*dT* were calculated during this 10-s period.

### Telemetric assessment

Arterial pressure and cage activity was measured simultaneously in awake, freely moving Thy1-aSyn and WT mice using radio telemetry transducers (TA11PA-C10; Data Sciences International Inc., St. Paul, MN, USA). Transmitter units were implanted subcutaneously along the mouse’s ventral side under the ketamine, xylazine, and buprenorphine anesthesia. The transmitter blood pressure catheter was fed over the shoulder and inserted into the left carotid artery under sterile surgical conditions (30). Data recording from the mice began immediately following implantation via an antenna receiver under the cage connected to a computer system. Pressure waveforms and activity data were collected, analyzed, and displayed with the telemetry software. For all studies, 20 s of data were collected every 10 min for the duration of the study. Data waveforms and parameters were analyzed with the DSI analysis program (ART 4.0) to determine differences in systolic, diastolic, and mean arterial pressures plus cage activity and HRs between Thy1-aSyn and WT mice during the entire light-dark diurnal cycle. Data for the hours surrounding the weekly scheduled cage changing were excluded from analysis. The data were averaged at the same time of day to create “foldagrams” to reveal any variances (dysfunction) and compare changes between groups under baseline conditions (30).

We tested tonic vagal release of acetylcholine at the muscarinic receptor of cardiac pacemaker cells with the muscarinic antagonist atropine [1 mg/kg, ip; (31)] administered to both Thy1-aSyn and WT mice. We documented blood pressure and baroreceptor reflex induced HR changes after phenylephrine [3 mg/kg, ip; (32)] and sodium nitroprusside [1 mg/kg, sc; (33)]. Each drug was administered at the same time of day at least 2 days apart to allow for complete drug clearance from the ip injection. Saline was used as the vehicle and administered before each drug injection. Blood pressure and HR were recorded continuously in the home cage, following saline, and following drug administration.

### Data expression and statistics

**Figure 1:**
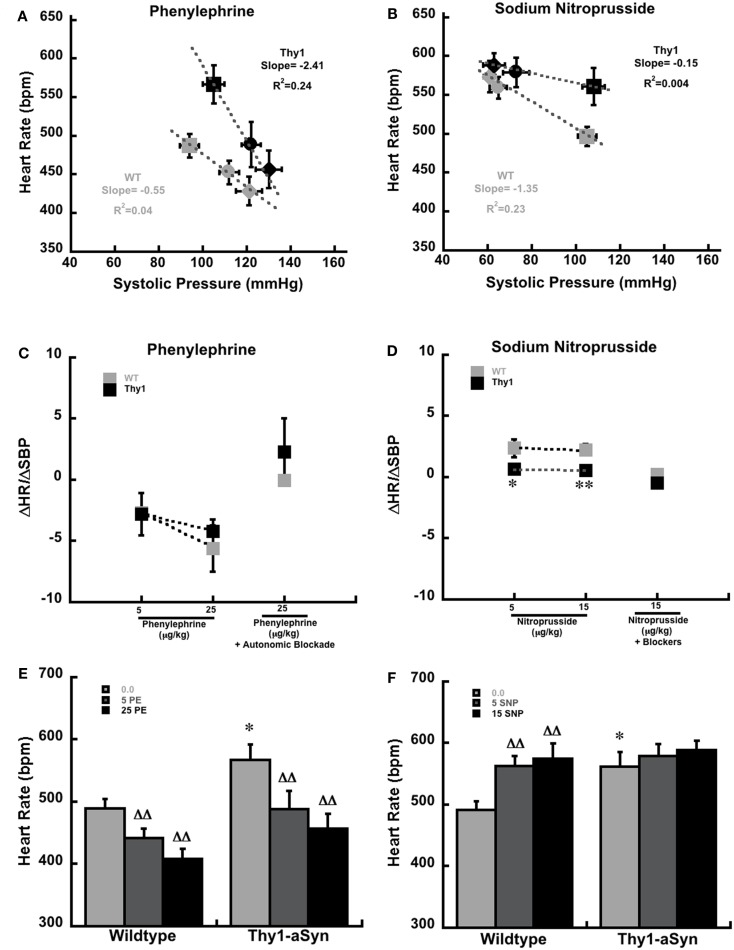
**Systolic pressure and heart rate at baseline (squares) and in response to phenylephrine (A) and sodium nitroprusside (B) in anesthetized WT (gray; *n* = 9) Thy1-aSyn (Black; *n* = 9) mice at 9–12 months of age**. Mice received 5 μg (circles) and 25 μg (diamonds) of intravenous phenylephrine and 5 μg (circles) and 15 μg (diamonds) of intravenous sodium nitroprusside. The slope and *R*^2^ values were derived from four separate regression equations, based on all available pairs of systolic blood pressure (mm/Hg) and heart rate (beats per minute) scores for each condition and drug, rather than the condition means and standard errors that are depicted in the plot. The baroreceptor sensitivity index (ΔHR/ΔSBP) following serial doses of phenylephrine alone and phenylephrine (25 μg/kg) following autonomic blockade with glycopyrrolate and propanolol **(C)**. ΔHR/ΔSBP following serial doses of sodium nitroprusside alone and sodium nitroprusside (15 μg/kg) following autonomic blockade with glycopyrrolate and propanolol **(D)**. *, **Represents *p* < 0.05, 0.01, respectively compared to WT at the same dose. Heart rate measurements at baseline (0.0 μg/kg) and following serial doses of phenylephrine [5 and 25 μg/kg; **(E)**]. Heart rate measurements at baseline (0.0 μg/kg) and following serial doses of sodium nitroprusside [5 and 15 μg/kg; **(F)**]. *Represents *p* < 0.01 compared to WT at same dose, DD represents *p* < 0.01 compared to baseline (0.0) within same genotype (Student’s *t*-test and mixed design ANOVA followed by Fisher’s LSD).

The hemodynamic experiment plots (Figures 1A–F) depict the baseline and acute post-drug pressure and HR data pairs as means and standard errors, computed as a function of condition, based on the nine mice in each experimental cell. The slope and *R*^2^ statistics were derived with standard bivariate linear regression analyses, determined from the raw systolic and beats per minute data pairs, as a function of the genotype × drug conditions, but collapsed across the three doses of each drug (i.e., *n* = 27 for each genotype × drug cell). These and the chronic telemetric data are also expressed as the ratio of the change in HR in BPM to the change in systolic blood pressure in mmHg, or ΔHR/ΔSBP. For the dobutamine challenge test and cardiac contractility experiments data are expressed as left ventricular +*dP*/*dT*_max_ and −*dP*/*dT*_min_. A mixed design ANOVA was used to compare genotypes and dose of drug in the hemodynamic and contractility experiments. Student’s *t*-test or Mann–Whitney *U* were applied to analyze morphometry parameters and arterial telemetry data between Thy1-aSyn and WT mice. Fisher’s Least Significant Difference was used for *post hoc* analyses. All statistics were calculated with GB-Stat software (Dynamic Microsystems, Inc., Silver Spring, MD, USA, 2000). The level of significance was set at *p* < 0.05.

## Results

### Morphometry parameters

Morphometry measures included measurements of body, heart, liver, and lung weights, and tibia length in Thy1-aSyn and WT mice (Table 1). Comparisons between WT and Thy1-aSyn mice using Student’s *t*-test showed that Thy1-aSyn mice weighed significantly less than WT mice, a difference we reported previously in this mutant line (19). While there were no significant differences in any of the other measures, heart and liver weights tended to be smaller in Thy1-aSyn mice but the difference did not reach significance (*p* = 0.08 and *p* = 0.06, respectively).

**Table 1:** **Morphometry parameters in Thy1-aSyn and wildtype mice at 9–12 months of age**.

	Wildtype	Thy1-aSyn
Body weight (g)	42.12 ± 2.60	31.99 ± 2.36*
Heart weight (mg)	203.43 ± 13.42	172.93 ± 9.75
Lung weight (mg)	203.98 ± 8.35	208.92 ± 27.94
Liver weight (g)	1.45 ± 0.17	1.02 ± 0.12
Tibia length (mm)	18.10 ± 0.21	18.02 ± 0.15
Heart weight/body weight	4.86 ± 0.23	5.50 ± 0.24
Heart weight/tibia length	11.24 ± 0.74	9.59 ± 0.52

*p < 0.05 compared to wildtype.

### Baseline heart rate and systolic blood pressure

Baseline systolic blood pressure measurements did not differ between conscious or anesthetized WT and Thy1-aSyn mice (*p* > 0.05). However, baseline HR measures were significantly different between genotypes under anesthesia during the acute hemodynamic studies (Table 2); Thy1-aSyn mice had a higher HR compared to WT mice (*p* < 0.05). In contrast, conscious WT and Thy1-aSyn mice equipped with arterial telemetry had comparable baseline HRs during both the light and dark cycles (Table 2).

**Table 2:** **Baseline heart rate and blood pressure (BP) in anesthetized and awake Thy1-aSyn and wildtype mice**.

Genotype	Age (m)		Cycle	Heart rate (beats/min)	Systolic BP (mmHg)
Wildtype	9–12	Anesthetized	Light	489 ± 14	97 ± 4
Thy1-aSyn	9–12	Anesthetized	Light	567 ± 25*	106 ± 5
Wildtype	3–5	Awake	Light	475 ± 7	108 ± 3
Thy1-aSyn	3–5	Awake	Light	498 ± 24	111 ± 3
Wildtype	3–5	Awake	Dark	544 ± 13	127 ± 1
Thy1-aSyn	3–5	Awake	Dark	589 ± 27	129 ± 3

*p < 0.05 compared to wildtype.

### Hemodynamic assessment

The baroreflex response to the injection of phenylephrine and sodium nitroprusside in anesthetized Thy1-aSyn and WT mice are plotted in Figures 1A,B. Though the plots show averaged pressure and heart values for each condition for clarity, the individual baseline systolic pressures were measured over a wide range of values from 48 to 159 mmHg in WT mice and from 41 to 157 mmHg in Thy1-aSyn mice. Linear regression analysis from the raw data pairs (*n* = 27 pairs per study) are drawn through the averaged data points under each condition in Figures 1A,B. These data are also plotted as the ΔHR/ΔSBP score (baroreflex sensitivity index) in Figures 1C,D. These data clearly show that phenylephrine produced a similar increase in systolic blood pressure and reflex decrease in HR in WT and Thy1-aSyn mice resulting in ΔHR/ΔSBP scores that did not differ between genotypes over this range of baseline pressures (Figure 1C). As expected with combined β-adrenergic and muscarinic receptor blockade, the ΔHR/ΔSBP response to phenylephrine following β-adrenergic and muscarinic blockade also did not differ between genotypes (Figure 1C). In contrast, the response to sodium nitroprusside in WT mice showed the characteristic decrease in systolic blood pressure and increased HR, while in Thy1-aSyn mice systolic blood pressure decreased but HR remained close to their initial baseline values regardless of dose (Figures 1B,D). Here, Thy1-aSyn regression slope was very shallow and ΔHR/ΔSBP scores were significantly lower compared to WT mice (*p* < 0.05). The ΔHR/ΔSBP response to sodium nitroprusside following β-adrenergic and muscarinic blockade did not differ between genotypes (Figure 1D).

Analysis of HR in WT and Thy1-aSyn mice showed phenylephrine caused similar HR decreases in both genotypes following administration of phenylephrine [Figure 1E; *F*(1,16) = 4.33, *p* > 0.05 for genotype and *F*(2,32) = 39.47, *p* < 0.01 for drug dose]. However, analysis of HR following sodium nitroprusside revealed that WT but not Thy1-aSyn showed significant increases in HR after each dose [Figure 1F; *F*(1,16) = 1.69, *p* > 0.05 for genotype and *F*(2,32) = 25.65, *p* < 0.01 for drug dose, and *F*(2,32) = 7.34, *p* < 0.05 for genotype × drug dose interaction].

### Dobutamine challenge

**Figure 2:**
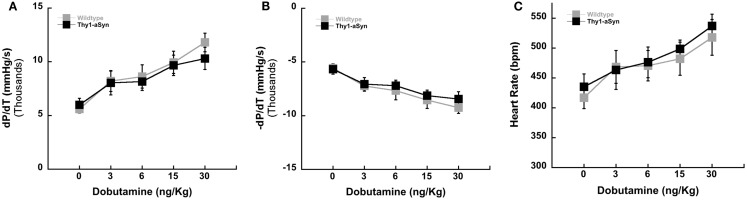
**Left ventricular contractility in WT (*n* = 7) and Thy1-aSyn (*n* = 7) at 9–12 months of age**. Left ventricular positive *dP*/*dT* [contractility; **(A)**], left ventricular negative *dP*/*dT* [relaxation; **(B)**], and heart rate following administration of the β-adrenergic agonist dobutamine [**(C)**; mixed design ANOVA].

Left ventricular inotropy (contraction) and lusitropy (relaxation) (±*dP*/*dT*) were measured in WT and Thy1-aSyn mice at 9–12 months of age (Figure 2). Serial infusions of the β-adrenergic agonist dobutamine resulted in similar left ventricular contractility (+*dP*/*dT*; Figure 2A) and relaxation (−*dP*/*dT*; Figure 2B) between WT and Thy1-aSyn mice (*p* > 0.05). HR responses were also similar between genotypes (*p* > 0.05; Figure 2C).

### Telemetric assessment

**Figure 3:**
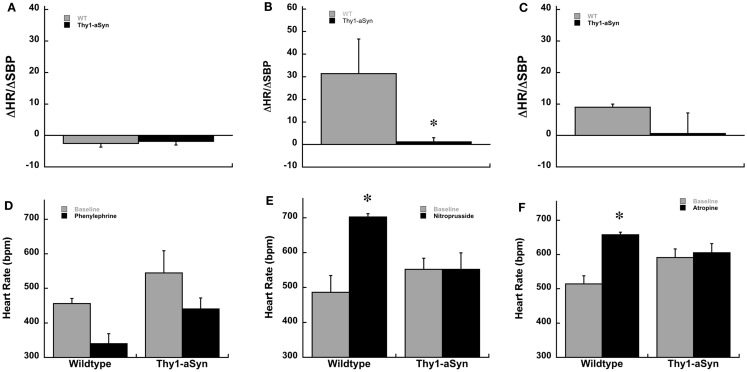
**Baroreceptor sensitivity, ΔHR/ΔSBP, in awake Thy1-aSyn (*n* = 3–5) and WT (*n* = 3–4) mice at 3–5 months following systemic administration of phenylephrine (A), sodium nitroprusside (B), and atropine (C)**. Heart rate responses following systemic phenylephrine **(D)**, sodium nitroprusside **(E)**, and atropine **(F)**. *Represents *p* < 0.05 compared to WT, Δ represents *p* < 0.05 compared to baseline (0.0 mg/kg) within same genotype (Mann–Whitney *U* and mixed design ANOVA followed by Fisher’s LSD).

Because Thy1-aSyn mice had a higher HR at baseline under anesthesia we wanted to determine if the differences we observed were due to an abnormal response to the anesthesia or to impaired baroreflex. Therefore, a separate cohort of Thy1-aSyn and WT mice were implanted with arterial telemetry transducers. Due to poor post surgery survival at the 9- to 12-month age in Thy1-aSyn mice the separate cohort of mice for telemetry was younger in age, 3–5 months. Similar to the hemodynamic experiment under anesthesia, conscious mice were challenged pharmacologically with intraperitoneal phenylephrine and nitroprusside (ip). In addition the response to muscarinic blockade was measured using atropine. At 3–5 months of age baseline HR and systolic blood pressure did not differ between WT and Thy1-aSyn mice. Similar to the anesthetized experiment WT and Thy1-aSyn mice displayed comparable responses to phenylephrine and nitroprusside over a similar range of systolic pressures. There was no difference between the genotypes in ΔHR/ΔSBP or HR scores following phenylephrine injections (*p* > 0.05; Figures 3A,D). However, in response to sodium nitroprusside Thy1-aSyn mice once again showed a blunted HR response while WT showed characteristic tachycardia. Thy1-aSyn ΔHR/ΔSBP scores were significantly decreased compared to WT (*p* < 0.05; Figure 3B). Analysis of HR alone showed that HR significantly increased in WT but not in Thy1-aSyn mice following nitroprusside (*p* < 0.05; Figure 3E). In response to muscarinic blockade ΔHR/ΔSBP scores did not significantly differ between genotypes however, analysis of HR alone showed that HR significantly increased in WT but not in Thy1-aSyn mice following atropine (Figures 3C,F). The activity response did not significantly differ between genotypes in any of the drug conditions (Student’s *t*-test, *p* > 0.05).

## Discussion

Baroreflex failure has been reported in both sporadic and alpha-synuclein associated familial forms of PD (4, 15). While cardiovascular autonomic dysfunction is well established in PD, little is known about the underlying cause of the dysfunction. Until recently, there has been a lack of basic research on the non-motor impairments associated with, and often present very early in humans with PD. However, with the potential early occurrence of many of the non-motor symptoms, a keen interest in these symptoms as possible early disease biomarkers has developed. For example, impaired HR variability observed in REM sleep behavior disorder may be an early indicator of PD (7). In the present study we show that overexpression of human alpha-synuclein in mice leads to impaired baroreflex control of HR prior to changes in striatal dopamine content and reminiscent of the baroreflex dysfunction observed in PD (1, 2). These findings are important because they provide a foundation for future studies aimed at identifying brain structures and pathological mechanisms associated with cardiovascular autonomic dysfunction in PD. In addition, these results underscore the usefulness of Thy1-aSyn mice in the study of early non-motor symptoms associated with PD.

Overall, with the exception of body weight, morphometry measures did not statistically differ between genotypes. We have previously reported decreased body weight in Thy1-aSyn compared to WT mice at 12 months of age without differences in overall food intake (19). We did however note a trend for heart and liver weights to be decreased in Thy1-aSyn mice.

The most profound functional differences were observed in response to administration of the vasodilator sodium nitroprusside. Here, anesthetized WT mice displayed the characteristic tachycardic response to the sudden drop in blood pressure produced by sodium nitroprusside, over a wide range of initial baseline values. But the Thy1-aSyn mice showed no significant change in HR during an equivalent transient decrease in systolic blood pressure suggesting impairments in autonomic modulation of cardiac function in Thy1-aSyn mice at an early age, before reductions in striatal dopamine content are observed (23). During these experiments it was noted that Thy1-aSyn mice appeared more sensitive than WT to the anesthetic combination of ketamine, xylazine, and buprenorphine. This is consistent with a recent study in the same Thy1-aSyn mouse line where the authors report increased sensitivity to the common rodent anesthetic xylazine (22). Due to this increased sensitivity it is possible that the HR differences observed in the Thy1-aSyn mice could be due in part to the anesthesia and not impaired baroreflex function. Therefore, we performed an additional set of experiments where WT and Thy1-aSyn mice were equipped with arterial telemetric transducers to allow HR and SBP measurements in conscious/awake mice. In this experiment we found that baseline HR and SBP were not different between genotypes suggesting the difference seen in anesthetized mice may have been a consequence of abnormal response to the anesthesia, or only develop at a later age. However, HR and SBP responses to phenylephrine and sodium nitroprusside were similar to what we observed in the previous experiments under anesthesia. In Thy1-aSyn mice sodium nitroprusside administration resulted in a significant decrease in SBP and a blunted HR response. Typically, during a drop in blood pressure efferent sympathetic outflow is increased (34, 35). Thus, in the present study the blunted HR response to sodium nitroprusside suggests the sympathetic component of the baroreflex is impaired in Thy1-aSyn mice over a wide range of ages. In PD patients with or without orthostatic hypotension, a similar blunted HR increase has been reported in response to the feet down tilt maneuver and in the Valsalva maneuver (5, 36, 37). It has been proposed that the combination of baroreflex failure and widespread noradrenergic denervation contributes to the development of orthostatic and post-prandial hypotension and supine hypertension commonly seen in PD (38).

Adrenergic supersensitivity in response to sympathetic denervation has been reported in patients with PD (39). Therefore, similar to Nakamura et al. (39) we tested left ventricular contractility of the heart in response to administration of the β-adrenergic agonist dobutamine in WT and Thy1-aSyn mice. There were no significant differences in inotropy or lusitropy between WT and Thy1-aSyn mice indicating no evidence of receptor supersensitivity. This is consistent with a recent finding showing no differences in contractility in patients in the early stages of PD (40).

Acetylcholine released from parasympathetic nerves reduces HR by binding primarily to M2 subtype muscarinic receptors on sinoatrial nodal cells in the heart. Blockade of M2 muscarinic receptors results in an increase in HR (31). In the present study WT but not Thy1-aSyn mice showed a significant increase in HR in response to the systemic muscarinic antagonist atropine. In addition, although not statistically significant (*p* = 0.08), WT mice showed a mean increase in HR in response to glycopyrrolate while Thy1-aSyn mice showed on average a decrease in HR in response to the peripheral muscarinic antagonist. The abnormal response to atropine in Thy1-aSyn mice is similar to a recent study measuring HR variability parameters in mice that express A53T mutant human alpha-synuclein under the Thy1 promoter. Here, the authors show increased baseline HR and reduced change in HR in response to systemic atropine in A53T compared to WT mice suggesting parasympathetic dysfunction in the mutant mice (11). However in contrast, mice with A53T or A30P mutations in alpha-synuclein generated using PAC transgenesis showed no alterations in HR variability parameters at 12 months of age, perhaps due to the low level of expression of the transgenic protein (10). The abnormal cardiovascular response to atropine taken together with the impaired HR response to sodium nitroprusside and previous work showing gastrointestinal dysfunction in Thy1-aSyn mice suggest impairment of both sympathetic and parasympathetic systems in Thy1-aSyn mice (19, 20). Thus, the Thy1-aSyn mice show a potentially broader range of autonomic anomalies than previously reported in other mouse models of synucleinopathy (10, 11).

The pathological mechanisms underlying cardiovascular autonomic dysfunction in PD remain unclear. In PD sympathetic denervation is observed in patients with and without orthostatic hypotension and has been reported to develop before and after the diagnosis of PD (4, 38, 41). In addition to sympathetic denervation alpha-synuclein containing Lewy bodies can be observed in cardiac and brain regions important for sympathetic regulation of blood pressure and HR in humans suggesting that alpha-synuclein pathology may contribute to cardiovascular autonomic anomalies commonly observed in PD (12–14). In the brain of Thy1-aSyn mice human alpha-synuclein mRNA expression is high throughout the brainstem and proteinase K-resistant alpha-synuclein inclusions develop in the locus coeruleus and substantia nigra (16, 17). In a recent study in the same line of Thy1-aSyn mice human alpha-synuclein protein was observed in the ventricular and atrial walls of the heart localized within noradrenergic fibers (22). Thus, it is unclear at this point whether the functional impairments are associated with either peripheral or central pathology or a combination of both. Functional deficits in central noradrenergic and dopaminergic systems have been observed in this mouse line (23, 24, 42, Maidment and Chesselet, unpublished observations) and dysregulation of central noradrenergic and dopaminergic systems has been shown to alter baroreceptor function in rodents suggesting central catecholamine system dysfunction could be a contributing factor (8, 27). Sodium nitroprusside, in addition to its peripheral vasodilator actions, is also known to activate central noradrenergic neurons in the locus coeruleus (43). Interestingly, the most profound impairment in Thy1-aSyn mice was a lack of nitroprusside-induced tachycardia, which was shown in both anesthetized and awake conditions. However, further research is needed to determine the exact contribution of peripheral and central noradrenergic and dopaminergic dysfunction in the autonomic regulation of blood pressure and HR in Thy1-aSyn mice.

## Conclusion

The present study shows that Thy1-aSyn mice, a model of synucleinopathy, have impaired baroreflex control of HR analogous to what is observed in patients with the synucleinopathy PD (5, 36, 37). Furthermore, the cardiovascular autonomic dysfunction detected in the Thy1-aSyn mice occurs prior to dopaminergic alterations in the striatum. Similarly in PD cardiovascular autonomic dysfunction is also reported to occur early, before the onset of overt motor symptoms. While the mechanisms contributing to cardiovascular anomalies in PD remain to be established the Thy1-aSyn mice provide a novel genetic model in which to study the contribution of alpha-synuclein pathology to autonomic dysfunction in PD.

## Conflict of Interest Statement

The authors declare that the research was conducted in the absence of any commercial or financial relationships that could be construed as a potential conflict of interest.
